# Variations in Visual Search Behavior Before Freezing of Gait Episodes: A Case Series

**DOI:** 10.7759/cureus.80892

**Published:** 2025-03-20

**Authors:** Yuki Kondo, Daisuke Muroi

**Affiliations:** 1 Department of Physical Rehabilitation, National Center of Neurology and Psychiatry, Tokyo, JPN; 2 Department of Rehabilitation Sciences, Faculty of Healthcare Sciences, Chiba Prefectural University of Health Sciences, Chiba, JPN

**Keywords:** case-series, freezing of gait, gaze behavior, progressive supranuclear palsy, visual attention, visuomotor

## Abstract

Reports on the relationship between freezing of gait (FoG) and visual search behavior in Parkinsonian syndrome remain limited. This study aimed to examine gaze behavior in three patients with progressive supranuclear palsy during doorway walking trials. Contrary to previous case reports, substantial individual variability was observed in gaze fixation patterns and blink rates during the FoG episodes. In conclusion, the relationship between visual search behavior and freezing is more complex than previously understood.

## Introduction

Freezing of gait (FoG), defined as "a brief, episodic absence, or marked reduction of forward progression of the feet despite the intention to walk" [1], is a complex motor symptom that commonly occurs in approximately 50% of patients with Parkinson’s disease (PD) and Parkinsonian syndromes (progressive supranuclear palsy (PSP), corticobasal syndrome (CBS)) [2,3]. This phenomenon is influenced by various neural and physiological factors that significantly affect the patient’s quality of life [4-6].

Previous studies have reported significant findings regarding visual search behaviors in individuals with a FoG [7,8]. Visual search behavior refers to the pattern of eye movements used to gather information from the environment, which is crucial for planning and executing safe locomotion. Gaze fixation, which refers to the maintenance of visual gaze on a single location, has been shown to differ between freezing and non-freezing episodes. In one study, a single participant fortuitously demonstrated FoG episodes during experimental trials, allowing for a unique comparison between trials with and without freezing. Regarding the trials with freezing episodes, individuals with FoG exhibited gaze fixation allocation towards obstacles and the immediate floor area while demonstrating markedly reduced gaze fixations on prospective walking paths. Conversely, during trials without freezing episodes, these individuals displayed decreased gaze fixation duration on obstacles and the immediate floor area while exhibiting a more proportionate gaze distribution along prospective walking paths [8]. These observations suggest that maladaptive gaze fixation patterns may impair the conscious motor control required for safe ambulation, potentially leading to FoG episodes. This aligns with clinical impressions in which inappropriate visual search behavior is frequently observed during freezing episodes.

FoG episodes are typically difficult to observe under experimental conditions and rely predominantly on patient self-reporting [9]. Notably, previous studies involving 21 participants showed that FoG episodes were directly observed in only one participant under experimental conditions [7,8]. The remaining 20 participants reported experiencing FoG in their daily lives; however, no freezing episodes were observed during the experiments.

We specifically focused on patients with PSP for this study because they often exhibit more severe and frequent FoG episodes than those with PD, making them particularly suitable subjects for investigating visual search behavior during freezing episodes. Additionally, PSP is characterized by distinctive oculomotor abnormalities, including impaired vertical gaze, square wave jerks, and slowed saccades, which may provide unique insights into the relationship between gaze control and gait dysfunction. Studying this population offers an opportunity to observe freezing in a more predictable manner within experimental settings, potentially revealing mechanisms that might be applicable across various Parkinsonian syndromes.

We observed FoG episodes during visual search behavior experiments in three participants with PSP, enabling comparisons between trials with and without freezing. These observations provide a rare opportunity to examine this challenging phenomenon in a controlled setting. This case-series study aimed to present three cases and provide new insights into the relationship between visual search behavior and FoG episodes, which differ from previous findings.

## Case presentation

Three ambulatory patients with PSP presented with gait difficulties owing to FoG. All patients experienced FoG, confirmed either through a "Yes" response to the first question of the New Freezing of Gait Questionnaire (NFOGQ) ("Did you experience any freezing of gait episodes within the last month?") [10], and through direct observation of freezing episodes during laboratory assessment [11]. Although we initially recruited patients with various Parkinsonian syndromes, only three exhibited observable FoG during the experimental tasks. The detailed clinical findings for each case are summarized in Table 1 (see Appendix A for descriptions of the clinical measures).

**Table 1:** Demographic and clinical characteristics of participants. MMSE: Mini-Mental State Examination; MoCA-J: Montreal Cognitive Assessment-Japanese version; FAB: Frontal Assessment Battery; H&Y: Hoehn and Yahr; PSP: progressive supranuclear palsy; NFOGQ: New Freezing of Gait Questionnaire; GES: Gait Efficacy Scale; GSAP: Gait-Specific Attentional Profile

Characteristics	Case A	Case B	Case C
Age (years)	76	68	70
Sex	Male	Male	Female
Height (cm)	172.3	167.8	158.2
Weight (kg)	71.2	59.0	43.0
Duration time (years)	16	9	6
Cognitive Function			
MMSE	28	30	30
MoCA-J	26	25	26
FAB	9	16	18
Disease Severity			
H&Y stage	III	IV	III
PSP Rating Scale			
I. History/daily activities	3	5	4
II. Mental	1	2	1
III. Bulbar	4	9	3
IV. Ocular motor	7	7	6
V. Limb motor/gait	4	9	5
NFOGQ			
Q1: Screening question about freezing episodes experience	1	1	1
Q2: Frequency of freezing episodes	1	3	2
Q3: Frequency during turning	3	1	1
Q4: Duration of longest freezing episode during turning	4	3	3
Q5: Frequency when initiating first step	3	4	4
Q6: Duration of longest freezing episode when initiating first step	4	4	3
Q7: Disturbance to daily walking	3	4	4
Q8: Impact on feelings of insecurity and fear of falling	1	3	3
Q9: Impact on daily activities	0	2	2
GES	84	77	42
GSAP Scores			
Anxiety	4	3	6
Internal focus	8	12	12
Rumination	6	6	7
Processing inefficiency	6	4	6

Protocol

The participants completed six doorway walking trials. The doorway width was adjusted to 100% of each participant's shoulder width, as FoG is known to be more frequent when walking through narrow spaces [12,13]. Prior to each trial, the participants were instructed to stand still at a marked position 5 m from the doorway for 30 seconds (standing task). During this standing period, participants were asked to imagine passing through a doorway without colliding. The standing task was incorporated to examine the participants' offline motor planning before actual gait initiation. This approach allowed us to understand how individuals process and evaluate spatial information in the stationary state, potentially revealing the anticipatory visual search strategies used for movement planning before locomotion occurs. After the standing period, the participants were asked to walk at a self-determined pace and pass through the doorway without collision (walking task).

Freezing episodes were analyzed using video recordings. FoG was defined as "brief, episodic absence, or marked reduction of forward progression of the feet despite the intention to walk" [1]. A trial was classified as a freezing trial when a participant experienced FoG at any point until they completed the passage through the doorway. Freezing episodes were independently assessed by two physical therapists who specialized in movement disorder rehabilitation and had 10 and 20 years of clinical experience. Since our assessment focused on the presence or absence of FoG rather than its duration, there was minimal disagreement between evaluators. When disagreements did occur, they were readily resolved by consensus through discussion [14].

**Figure 1:**
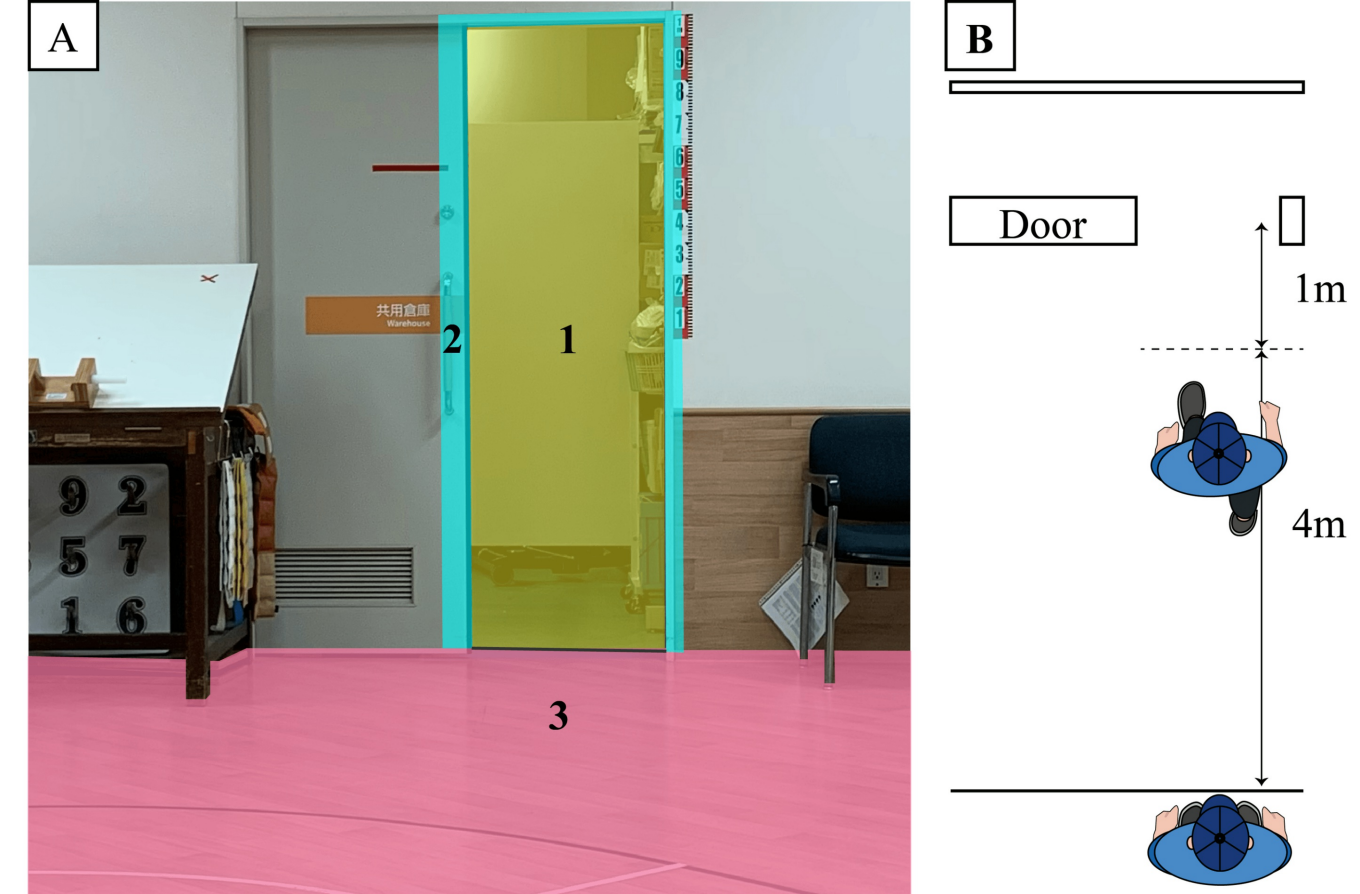
Areas of interest and task environment. (A) Areas of interest are displayed using different colors: 1 = Doorway (yellow) - the path through the door opening; 2 = Door frame (light blue) - the structural elements of the doorway; 3 = Floor (pink) - the walking surface directly in front of the participant. These areas were tracked during both standing and walking tasks to analyze gaze fixation patterns. (B) An overhead schematic diagram of the task setup. The diagram shows the spatial layout of the experimental environment viewed from above, illustrating the positions of key elements in the task: the participant's starting position (5 m from the doorway), the walking path, and the doorway with width adjusted to 100% of each participant's shoulder width. Credit (B): Image created by the authors.

The participants’ visual search behavior was recorded using an eye-tracking system (EMR-9, NAC Image Technology Inc., Tokyo, Japan) during both the standing and walking tasks. The system was operated at a sampling frequency of 60 Hz and a resolution of 0.1 degrees. Before data collection, the eye tracker was calibrated using a 9-point calibration procedure for each participant. Fixations were classified into three distinct areas of interest: walkways, doorways, and door frames (Figure 1). We analyzed the gaze fixation duration during gait, which has previously been used as a primary measure in FoG research [7,8]. To provide additional insights into visual search behavior during FoG, we analyzed three parameters that have not been previously quantified in FoG research: gaze fixation duration during standing tasks, blink count, and gaze fixation switches between areas of interest. For both standing and walking tasks, we analyzed gaze fixation duration (total time spent fixating on each area of interest, where gaze fixation was defined as when the eye marker remained at a point of gaze for more than 100 milliseconds), blink count, and gaze fixation switches (number of transitions between different areas of interest). Trials in which eye-tracking data were not properly recorded (e.g., loss of pupil detection) were excluded from the analysis. The visual search behavior parameters were analyzed separately for the standing (30 seconds) and walking periods (from the initiation of walking until one meter before the doorway, corresponding to the first four meters of the walking trajectory).

Based on previous studies [8], we hypothesized that during walking tasks, trials with FoG episodes would be characterized by increased gaze fixation duration on door frames (obstacles) or the immediate floor area, whereas non-freezing trials would show longer gaze fixation duration in the doorway space, reflecting a more anticipatory visual search strategy. This exploratory case series was designed to provide detailed observations of visual search behavior during FoG episodes, which are notoriously difficult to capture in laboratory settings [9]. Despite the small sample size (n=3), the case series approach allows for in-depth analysis of individual patterns that might be obscured in larger group-level analyses. Each case serves as its own control (freezing vs. non-freezing trials), enabling within-subject comparisons that control for individual variability in baseline gait and visual search characteristics.

This study was approved by the Institutional Review Board of the National Center of Neurology and Psychiatry (NCNP) (approval number A2022-012) and the Chiba Prefectural University of Health Sciences (approval number 2022-12) and was conducted in accordance with the guidelines of the Ethics Committee of the NCNP and the Declaration of Helsinki. All participants provided written informed consent before participation. This study was conducted at the NCNP. The eye-tracking system and technical support for visual search behavior analysis were provided by the Chiba Prefectural University of Health Sciences.

Results

Overview

**Figure 2:**
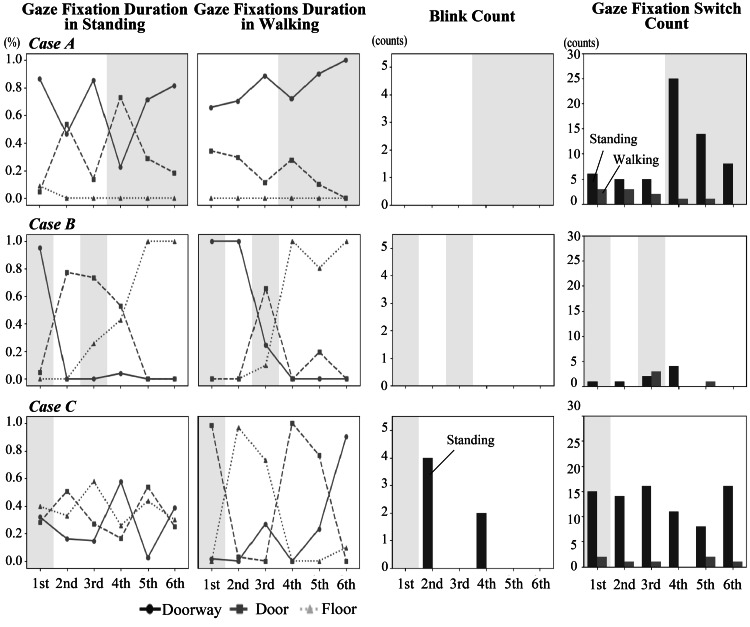
Visual search behavior parameters during standing and gait tasks in Cases A-C. The graphs show gaze fixation distribution (%), blink counts, and fixation switch counts during both standing and walking tasks across all six trials for each case. Shaded areas (gray) indicate trials where FoG occurred: Case A (trials 4-6), Case B (trials 1 and 3), and Case C (trial 1).

FoG episodes were observed across all cases: Case A experienced FoG during trials 4-6, Case B during trials 1 and 3, and Case C during trial 1. All FoG episodes occurred specifically during doorway passages. The relationship between FoG and the visual search behavior parameters is shown in Figure 2 and Table 2. In Figure 2, shaded areas highlight trials with FoG, allowing for direct visual comparison between freezing and non-freezing trials across multiple parameters. Table 2 provides the exact numerical values corresponding to each parameter, enabling quantitative analysis of the patterns observed.

**Table 2:** Quantitative data of visual search behavior parameters corresponding to Figure 1. FoG: freezing of gait

Case	Trial	FoG occurrence	Duration of fixations (%)						Blink counts (counts)		Fixation switch count (counts)	
			Standing			Gait			Standing	Gait	Standing	Gait
			Doorway	Door	Floor	Doorway	Door	Floor				
A	1	No	86.7	4.5	8.8	65.8	34.2	0.0	0	0	6	3
	2	No	46.4	53.6	0.0	70.4	29.6	0.0	0	0	5	3
	3	No	85.5	13.7	0.0	88.7	11.3	0.0	0	0	5	2
	4	Yes	22.5	73.2	0.0	72.3	27.7	0.0	0	0	25	1
	5	Yes	71.4	28.6	0.0	90.0	10.0	0.0	0	0	14	1
	6	Yes	81.6	18.4	0.0	100.0	0.0	0.0	0	0	8	0
B	1	Yes	95.1	4.9	0.0	100.0	0.0	0.0	0	0	1	0
	2	No	0.0	77.4	0.0	100.0	0.0	0.0	4	0	1	0
	3	Yes	0.0	73.6	25.7	24.5	65.7	9.8	0	0	2	3
	4	No	4.0	53.0	42.9	0.0	0.0	100.0	2	0	4	0
	5	No	0.0	0.0	100.0	0.0	19.4	80.6	0	0	0	1
	6	No	0.0	0.0	100.0	0.0	0.0	100.0	0	0	0	0
C	1	Yes	32.0	28.3	39.7	1.6	98.4	0.0	0	0	15	2
	2	No	16.1	50.9	33.0	0.0	2.9	97.1	0	0	14	1
	3	No	14.8	27.2	58.0	26.8	0.0	73.2	0	0	16	1
	4	No	57.6	16.5	25.8	0.0	100.0	0.0	0	0	11	0
	5	No	2.7	53.7	43.6	23.3	76.7	0.0	0	0	8	2
	6	No	38.6	25.1	30.0	90.3	0.0	9.7	0	0	16	1

Case A

Medical History and Clinical Presentation

A 76-year-old male presented with a 16-year history of progressive gait difficulties. He was diagnosed with PSP and had been experiencing FoG episodes. His cognitive function tests showed: Mini-Mental State Examination (MMSE) 28/30, Japanese version of the Montreal Cognitive Assessment (MoCA-J) 26/30, and notably, Frontal Assessment Battery (FAB) 9/18, indicating relatively preserved general cognitive function but impaired frontal lobe function. His disease severity was rated as Hoehn and Yahr (H&Y) stage III.

Clinical Assessment

NFOGQ responses revealed a low frequency of freezing episodes (Q2: 1 point) with more pronounced freezing during turning (Q3: 3 points). Notably, he showed a low fear of falling due to FoG (Q8: 1 point) and minimal impact on daily living (Q9: 0 points). His Gait Efficacy Scale (GES) score was 84, indicating relatively high confidence in walking ability.

Visual Search Behavior and Freezing Episodes

During the experimental trials, FoG was observed specifically during trials 4-6. His visual search behavior showed distinctive patterns: maintained consistently high doorway gaze fixation (65.8-100%) across both FoG and non-FoG trials; minimal blink counts across all trials; and increased gaze fixation switches during standing in FoG trials (8-25 counts) compared to non-FoG trials (5-6 counts). The experimental setup of Case A is presented in Video 1.

**Video 1:** Eye-tracking experimental setup and results for Case A.

Case B

Medical History and Clinical Presentation

A 68-year-old male presented with a nine-year history of PSP. His cognitive assessments showed intact function with MMSE 30/30, MoCA-J 25/30, and FAB 16/18. His condition was classified as severe (H&Y stage IV), with significant impairment in both the bulbar and limb motor/gait domains of the PSP Rating Scale.

Clinical Assessment

NFOGQ showed frequent freezing episodes (Q2: 3 points) with a significant impact on daily activities (Q7: 4 points). His GES score was 77, indicating moderately high confidence in walking ability despite severe symptoms.

Visual Search Behavior and Freezing Episodes

FoG was observed during trials 1 and 3. His visual search behavior demonstrated predominant floor gaze fixations during non-FoG trials (80.6-100%); minimal blink frequency; and consistently low gaze fixation switch counts during gait (0-3 counts) across all trials. The experimental setup of Case B is presented in Video 2.

**Video 2:** Eye-tracking experimental setup and results for Case B.

Case C

Medical History and Clinical Presentation

A 70-year-old female presented with a six-year history of PSP. Her cognitive function was well-preserved with MMSE 30/30, MoCA-J 26/30, and FAB 18/18. Disease severity was rated as H&Y stage III.

Clinical Assessment

NFOGQ indicated moderate freezing frequency (Q2: 2 points) with a significant impact on gait initiation (Q5: 4 points). Notably, she showed a low GES score (42) and high anxiety scores in the GSAP, suggesting significant concerns about walking ability.

Visual Search Behavior and Freezing Episodes

FoG was observed only during trial 1. Her visual search behavior showed variable gaze fixation patterns across all areas of interest; minimal blink frequency; and consistent gaze fixation switch counts across all trials (11-16 counts during standing, 0-2 counts during walking). The experimental setup of Case C is presented in Video 3.

**Video 3:** Eye-tracking experimental setup and results for Case C.

Follow-Up and Outcomes

All participants continued their regular physical therapy sessions following the study.

## Discussion

The present case series provides valuable insights into the complex relationship between visual search behaviors and FoG in individuals with PSP. Our findings revealed substantial heterogeneity in visual search behaviors across participants, challenging the conventional understanding of the uniform relationships between visual search behaviors and freezing episodes.

A notable finding was the marked individual variability in gaze fixation patterns in the walking tasks during both freezing and non-freezing trials. A single-case observation from a previous study, in which freezing episodes occurred fortuitously during experimental trials, reported increased gaze fixation duration on obstacles and immediate floor area while showing markedly reduced gaze fixations on prospective walking paths during these episodes [8]. However, our case series revealed more complex patterns: while Case A maintained consistently high doorway gaze fixations (65.8-100%) regardless of FoG occurrence, Case B predominantly focused on the floor during non-freezing episodes (80.6-100%), and Case C exhibited variable gaze fixation patterns across areas of interest. These disparate patterns suggest that individuals adopt different visual search behaviors to navigate challenging environments, regardless of whether they experience freezing episodes. The significance of this case series lies in its demonstration of multiple distinct gaze fixation patterns rather than a single characteristic pattern associated with FoG. This heterogeneity suggests that the relationship between visual search behavior and FoG is more complex than previously understood, potentially reflecting individual adaptations to movement challenges and varying compensatory strategies.

The analysis of additional parameters further underscores this heterogeneity. Blink rates were significantly reduced across all cases (approximately 5.3 blinks per minute in a high task load and 5.9 blinks per minute in a low task load [15]). Blinking serves multiple functions in visual information processing and not only provides necessary eye lubrication but also plays a crucial role in the segmentation and processing of visual information through default mode network activation [16]. This network activation during blinks allows for momentary “mental breaks” that help organize and integrate incoming visual information. Therefore, the reduced blink frequency observed in our patients may represent a disruption In the natural information processing mechanism. When combined with the complex visual demands of navigation, the reduced ability to effectively segment and organize incoming visual information may contribute to cognitive overload [17], potentially triggering FoG episodes by disrupting information processing. The relationship between reduced blink rate and cognitive overload suggests that blink rate may serve as an additional marker of information processing capacity in individuals with FoG.

In addition, when combining gaze fixation switch patterns with other clinical indicators, our findings may be explained by four established theoretical models of FoG [18]: (1) the interference model, which involves the inability to process concurrent inputs; (2) the threshold model, in which motor deficits accumulate to cause breakdown; (3) the decoupling model, which represents the disconnection between motor programming and execution; and (4) the cognitive model, in which response conflict leads to indecision.

Case A exhibited an increased gaze fixation switch count during the FoG trials. The patient scored 9 points on the FAB, indicating relatively low frontal lobe function. Responses to the NFOGQ revealed a low fear of falling due to FoG (Q8) and a minimal impact on daily living (Q9), whereas the GES showed relatively high scores, indicating good gait self-efficacy. These findings collectively point to attention-related issues, particularly evident in increased distractibility during later trials. This pattern aligns with the model (1), the interference model of the FoG, which suggests that freezing episodes may result from difficulties in the simultaneous processing of multiple attention demands.

Case B demonstrated consistently low gaze fixation switch counts during gait (0-3 counts) across all trials. The patient's condition was classified as severe based on the H&Y Scale and Progressive Supranuclear Palsy Rating Scale (PSP Rating Scale), with high scores noted in both the bulbar and limb motor/gait domains. The high frequency of freezing episodes was also confirmed by the Q2 of the NFOGQ. Previous research indicated a common pathomechanism of gait freezing and speech disturbance, which aligns with the high scores in both domains observed in this study [19]. This pattern aligns with a combination of models (2) and (3), as severe motor symptoms and frequent freezing correspond to the threshold model's concept of accumulated motor deficits, while minimal gaze adaptation suggests impaired motor programming characteristics of the decoupling model.

Case C maintained relatively consistent gaze fixation switch counts (11-16 counts during standing, 0-2 counts during walking) despite experiencing FoG only in trial 1. The patient had low scores on the GES and notably high scores in the anxiety domain of the gait-specific attention profile. These findings suggest that the freezing episode in the first trial may have been triggered by a lack of confidence when facing a novel task, indicating the involvement of the model (4). The specific occurrence of FoG during a novel task, combined with anxiety and reduced self-efficacy, corresponded well with the cognitive model's emphasis on response conflict and decision-making challenges. Although we discussed our findings in relation to the four theoretical models, it should be noted that these models are not mutually exclusive but rather interrelated, making it impossible to fully explain FoG through a single model. The heterogeneity observed in our cases suggests that different theoretical models might better explain FoG manifestations in different individuals or that multiple mechanisms might operate simultaneously within the same individual. However, the gaze fixation switch counts during standing tasks may have been influenced by individual variations in how participants mentally rehearsed the doorway passage; some may have imagined a single passage, while others may have repeated the mental imagery multiple times.

Although we initially hypothesized that identifying FoG-specific visual search behaviors would inform rehabilitation strategies through intentional gaze guidance, our findings suggest that such an approach may be premature. Future research with larger sample sizes could help establish the generalizability of these findings and potentially identify distinct subtypes of visual search behavior associated with FoG, which could lead to more targeted and effective rehabilitation strategies.

The heterogeneity in visual search behaviors we observed challenges the conventional understanding of a uniform relationship between gaze patterns and FoG. While we have discussed our findings in relation to the four theoretical models of FoG, it is important to emphasize that these models are not mutually exclusive but rather complementary explanations of a complex phenomenon. Our case series suggests that different individuals may exhibit freezing episodes through different predominant mechanisms, which could explain the variability in associated visual search behaviors. For instance, Case A's increased gaze fixation switches during FoG trials aligned with the interference model, Case B's severe motor symptoms and minimal gaze adaptation corresponded with the threshold and decoupling models, and Case C's anxiety and freezing during a novel task reflected elements of the cognitive model. This interpretation supports a more personalized approach to understanding FoG, in which interventions might be tailored to address the specific mechanism most relevant to each individual. The observed heterogeneity may represent distinct subtypes of visual search behavior associated with FoG or individual adaptations to movement challenges, highlighting the complexity of the freezing phenomenon beyond disease-specific patterns.

This study has some limitations. A key limitation is the absence of a control group, either healthy controls or patients with other Parkinsonian syndromes. Without statistical comparisons to these populations, we cannot determine whether the observed visual search patterns are specific to PSP, common across Parkinsonian syndromes, or related to FoG independent of the underlying condition. In addition, we studied individuals with PSP rather than those with PD. Although current literature has not reported significant differences in FoG characteristics between these conditions, PSP typically presents with more severe oculomotor abnormalities than PD [20]. However, our eye-tracking data revealed that participants with PSP were able to shift their areas of interest throughout the task, suggesting that their gaze patterns were not completely fixed despite oculomotor impairments. While this difference in the underlying oculomotor function between the PD and PSP populations should be acknowledged, the dynamic nature of the visual search behavior observed in our PSP cases indicates that oculomotor deficits may not directly influence freezing episodes during doorway navigation. Nevertheless, given the documented difficulty in experimentally inducing FoG episodes [9], our ability to capture and analyze freezing events, regardless of the underlying conditions, represents a valuable contribution to the field. Our experimental paradigm differed from those of previous studies. While Vanegas-Arroyave et al. examined visual search behavior in typical clinical environments [7] and Hardeman et al. investigated obstacle crossing followed by doorway navigation [8], our study focused solely on doorway navigation. These methodological and environmental differences may have influenced the observed visual search behavior.

Furthermore, while our small sample size (n=3) limits the generalizability of findings, it enabled detailed within-subject analyses between freezing and non-freezing trials. This approach allowed us to control for individual variability in baseline gait and visual search characteristics, which may be obscured in larger group-level analyses. Future studies with larger samples will be needed to determine whether the heterogeneity we observed represents distinct subtypes of visual search behavior associated with FoG or simply reflects individual differences in compensatory strategies.

## Conclusions

Visual search behaviors during FoG episodes in our three cases with PSP differ from previously reported patterns and exhibit substantial individual variability. Although this heterogeneity makes it challenging to identify a single characteristic pattern, these observations expand our understanding of the relationship between visual search behaviors and the freezing phenomenon. The variability we observed may reflect different underlying mechanisms of FoG, as illustrated by our cases aligning with different aspects of established theoretical models (interference, threshold, decoupling, and cognitive). This exploratory case series highlights the need for future research with larger samples to determine whether this heterogeneity represents distinct subtypes of visual search behavior or individual compensatory strategies, potentially informing more personalized rehabilitation approaches.

## Appendices

Appendix A

Mini-Mental State Examination (MMSE)

The MMSE is widely used to measure cognitive impairment. It assesses arithmetic, memory, and orientation functions. Higher scores indicated better cognitive function.

Montreal Cognitive Assessment - Japanese Version (MoCA-J)

The MoCA-J is a cognitive screening tool designed to detect mild cognitive impairment. It assesses multiple cognitive domains, including attention, concentration, executive functions, memory, language, visuoconstructional skills, conceptual thinking, calculations, and orientation. Higher scores indicated better cognitive function.

Frontal Assessment Battery (FAB)

The FAB is a brief cognitive test designed to assess frontal lobe function, including conceptualization, mental flexibility, motor programming, sensitivity to interference, inhibitory control, and environmental autonomy. Higher scores indicated better frontal lobe function.

Hoehn and Yahr Scale (H&Y)

The H&Y scale was used to describe the progression of Parkinson’s symptoms. It ranges from stage 0 (no signs of disease) to stage 5 (wheelchair-bound or bedridden unless assisted). Lower stages indicate less severe symptoms.

Progressive Supranuclear Palsy (PSP) Rating Scale

The PSP rating scale was used to assess the severity of PSP, a neurodegenerative disease. It evaluates various symptoms, including history/daily activities and mental, bulbar, ocular, and limb motor/gait symptoms. Lower scores indicate less severe symptoms.

New Freezing of Gait Questionnaire (NFOGQ)

The NFOGQ is a self-report questionnaire designed to assess the freezing of gait in patients with Parkinson's. It evaluated the frequency and severity of freezing episodes. Lower scores indicated less severe freezing of gait.

Gait Efficacy Scale (GES)

The GES is used to assess individuals’ confidence in their ability to perform various walking activities without falling. It evaluates the perceived self-efficacy related to gait and balance. Higher scores indicated greater confidence in their walking ability.

Gait-Specific Attentional Profile (GSAP)

The GSAP is a comprehensive assessment tool that evaluates attentional demands of gait in patients with neurological disorders. It provides a detailed profile of a patient's attentional focus during walking. Varies based on specific subscales and metrics used, but generally, lower attentional demands indicate better gait function.

## References

[1] Nutt JG, Bloem BR, Giladi N, Hallett M, Horak FB, Nieuwboer A (2011). Freezing of gait: moving forward on a mysterious clinical phenomenon. Lancet Neurol.

[2] Zhang WS, Gao C, Tan YY, Chen SD (2021). Prevalence of freezing of gait in Parkinson's disease: a systematic review and meta-analysis. J Neurol.

[3] Tosserams A, Mazaheri M, Vart P, Bloem BR, Nonnekes J (2021). Sex and freezing of gait in Parkinson's disease: a systematic review and meta-analysis. J Neurol.

[4] Moore O, Peretz C, Giladi N (2007). Freezing of gait affects quality of life of peoples with Parkinson's disease beyond its relationships with mobility and gait. Mov Disord.

[5] Cronin P, Collins LM, Sullivan AM (2024). Impacts of gait freeze on quality of life in Parkinson's disease, from the perspectives of patients and their carers. Ir J Med Sci.

[6] Sharafkhah M, Moayedi F, Alimi N, Fini ZH, Massoudifar A (2024). Motor and non-motor predictors of freezing of gait in Parkinson's disease: a retrospective cohort study. J Bodyw Mov Ther.

[7] Vanegas-Arroyave N, Chen DF, Lauro PM, Norato G, Lungu C, Hallett M (2022). Where do Parkinson's disease patients look while walking?. Mov Disord.

[8] Hardeman LE, Kal EC, Young WR, van der Kamp J, Ellmers TJ (2020). Visuomotor control of walking in Parkinson's disease: exploring possible links between conscious movement processing and freezing of gait. Behav Brain Res.

[9] Mancini M, Bloem BR, Horak FB, Lewis SJ, Nieuwboer A, Nonnekes J (2019). Clinical and methodological challenges for assessing freezing of gait: future perspectives. Mov Disord.

[10] Nieuwboer A, Rochester L, Herman T, Vandenberghe W, Emil GE, Thomaes T, Giladi N (2009). Reliability of the new freezing of gait questionnaire: agreement between patients with Parkinson's disease and their carers. Gait Posture.

[11] Barthel C, Mallia E, Debû B, Bloem BR, Ferraye MU (2016). The practicalities of assessing freezing of gait. J Parkinsons Dis.

[12] Shine JM, Moore ST, Bolitho SJ, Morris TR, Dilda V, Naismith SL, Lewis SJ (2012). Assessing the utility of Freezing of Gait Questionnaires in Parkinson's Disease. Parkinsonism Relat Disord.

[13] Cowie D, Limousin P, Peters A, Hariz M, Day BL (2010). Insights into the neural control of locomotion from walking through doorways in Parkinson's disease. Neuropsychologia.

[14] Cowie D, Limousin P, Peters A, Hariz M, Day BL (2012). Doorway-provoked freezing of gait in Parkinson's disease. Mov Disord.

[15] Gilat M (2019). How to annotate freezing of gait from video: a standardized method using open-source software. J Parkinsons Dis.

[16] Nakano T, Kato M, Morito Y, Itoi S, Kitazawa S (2013). Blink-related momentary activation of the default mode network while viewing videos. Proc Natl Acad Sci U S A.

[17] Beck EN, Ehgoetz Martens KA, Almeida QJ (2015). Freezing of gait in Parkinson's disease: an overload problem?. PLoS One.

[18] Nieuwboer A, Giladi N (2013). Characterizing freezing of gait in Parkinson's disease: models of an episodic phenomenon. Mov Disord.

[19] Park HK, Yoo JY, Kwon M (2014). Gait freezing and speech disturbance in Parkinson's disease. Neurol Sci.

[20] Kassavetis P, Kaski D, Anderson T, Hallett M (2022). Eye movement disorders in movement disorders. Mov Disord Clin Pract.

